# Novel Neuroprotective Function of Apical-Basal Polarity Gene *Crumbs* in Amyloid Beta 42 (Aβ42) Mediated Neurodegeneration

**DOI:** 10.1371/journal.pone.0078717

**Published:** 2013-11-18

**Authors:** Andrew M. Steffensmeier, Meghana Tare, Oorvashi Roy Puli, Rohan Modi, Jaison Nainaparampil, Madhuri Kango-Singh, Amit Singh

**Affiliations:** 1 Premedical Program, University of Dayton, Dayton, Ohio, United States of America; 2 Department of Biology, University of Dayton, Dayton, Ohio, United States of America; 3 Center for Tissue Regeneration and Engineering at Dayton, University of Dayton, Dayton, Ohio, United States of America; Roswell Park Cancer Institute, United States of America

## Abstract

Alzheimer's disease (AD, OMIM: 104300), a progressive neurodegenerative disorder with no cure to date, is caused by the generation of amyloid-beta-42 (Aβ42) aggregates that trigger neuronal cell death by unknown mechanism(s). We have developed a transgenic *Drosophila* eye model where misexpression of human Aβ42 results in AD-like neuropathology in the neural retina. We have identified an apical-basal polarity gene *crumbs (crb)* as a genetic modifier of Aβ42-mediated-neuropathology. Misexpression of Aβ42 caused upregulation of Crb expression, whereas downregulation of Crb either by RNAi or null allele approach rescued the Aβ42-mediated-neurodegeneration. Co-expression of full length Crb with Aβ42 increased severity of Aβ42-mediated-neurodegeneration, due to three fold induction of cell death in comparison to the wild type. Higher Crb levels affect axonal targeting from the retina to the brain. The structure function analysis identified intracellular domain of Crb to be required for Aβ42-mediated-neurodegeneration. We demonstrate a novel neuroprotective role of Crb in Aβ42-mediated-neurodegeneration.

## Background

Alzheimer's disease (AD) is a progressive neurodegenerative disorder with no effective cure to date. AD is characterized by the progressive loss of neurons in the hippocampus and cortex causing decline in cognitive and behavioral functions eventually leading to the death of the patient [1], [2]. AD neuropathology is associated with two types of abnormal protein deposition in the human brain *viz.*: (1) neurofibrillary tangles (NFTs) containing hyperphosphorylated forms of a microtubule associated protein Tau, and (2) the accumulation of the amyloid-beta (Aβ42) peptide [1]–[8]. Aβ42 is generated by improper (β- and γ-) cleavage of the transmembrane receptor amyloid precursor protein (APP), as well as by mutations linked to familial AD that affect APP processing [9]. The abnormal cleavage of APP causes the protein to be 42 amino acids long (Aβ42), whereas, the normal length of the protein is 40 amino acids long (Aβ40) [1], [2], [7], [8]. The amyloid hypothesis suggests that Aβ42 forms protofibrils and fibrils. Accumulation of Aβ42 impairs basic cellular processes due to oxidative stress, misregulation of intracellular calcium, ER stress [10], and aberrant signaling through interaction with several receptors [3], [5], [6], [8], which results in the death of neurons [7]. Therefore, it is important to understand the mechanism underlying Aβ42 mediated cell death and neurotoxicity.

Since the genetic machinery and basic cell biological pathways are conserved from insects to humans, several animal models have been employed to model AD. Despite the immense amount of information available from modeling AD in animal models such as the mouse [2], [7] and the fruit fly [7], [11]–[15], the exact mechanism(s) mediating Aβ42-dependent cell death are yet to be determined. The fruit fly has been a model organism for human diseases for many years since nearly 70% of human disease genes are conserved in flies [16]. We have used a *Drosophila melanogaster* eye model to express the human Aβ42 peptide [3].

The *Drosophila* eye model has been extensively employed to investigate patterning, growth, and cell biological processes [7], [14]–[16]. The adult *Drosophila* compound eye develops from an epithelial bi-layer structure housed inside the larva called the eye-antennal imaginal disc, which gives rise to an eye, antenna and head cuticle of the adult fly [17]. A synchronous differentiation event in the developing third instar larval eye imaginal disc differentiates retinal precursor cells to photoreceptor neurons [18]. The eye imaginal disc metamorphose to a pupal retina which develops into the adult eye comprising of about 800 units called ommatidia [18]. Each ommatidium contains eight photoreceptors, pigment cells and several support cells. In the pupal retina, the extra undifferentiated cells are eliminated by programmed cell death (PCD) [19]. PCD is not observed during earlier stages of larval eye development, however, abnormal extracellular signaling due to inappropriate levels of morphogens may trigger cell death in the developing larval eye imaginal disc [20]. We have found that Aβ42 dependent cell death is mediated, in part, through activation of the JNK signaling pathway [3]. However, blocking the JNK signaling pathway does not completely rescue the Aβ42-dependent cell death [3]. Therefore, there may be other genetic components that remain to be identified.

**Figure 1:**
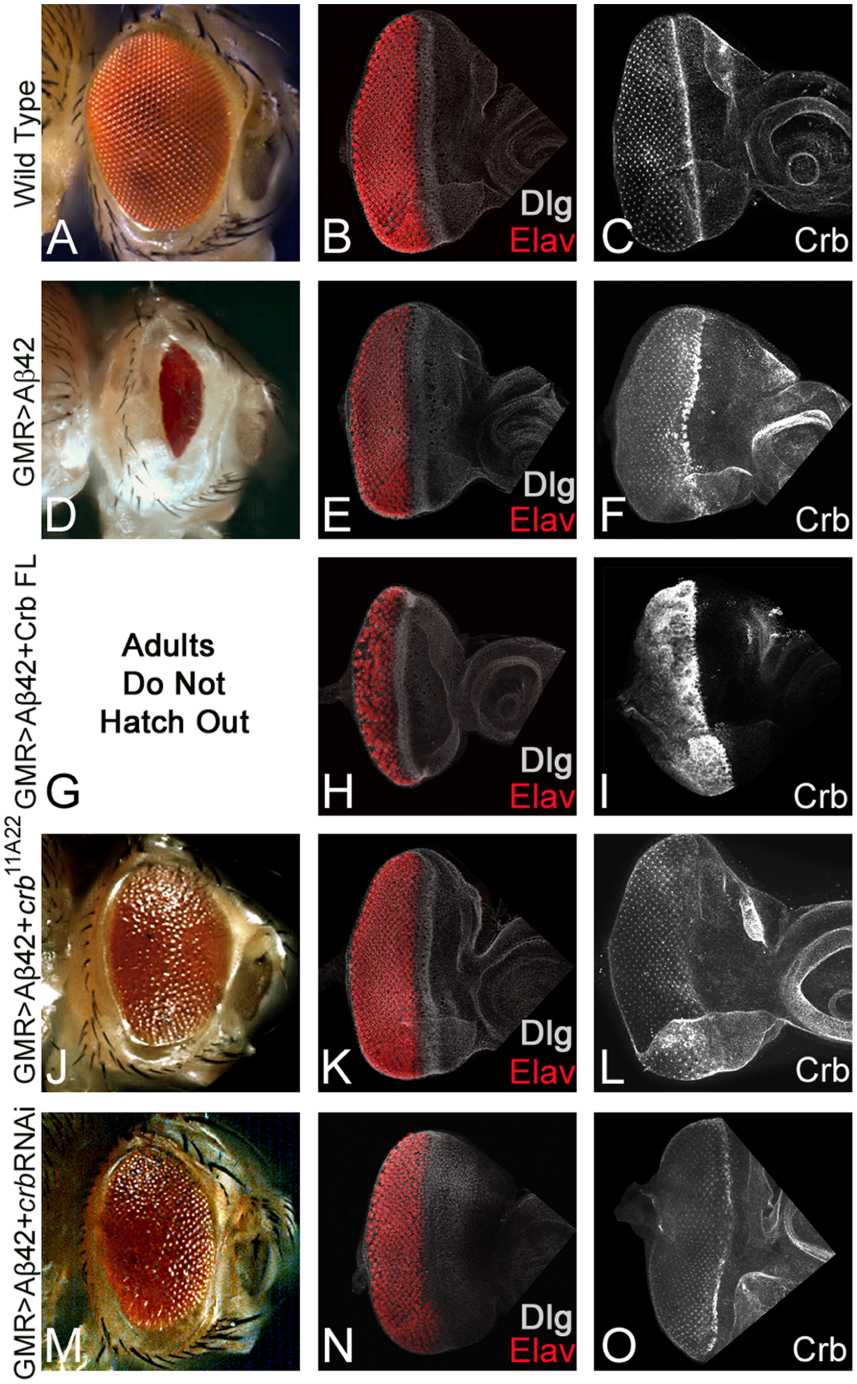
Levels of apical basal polarity gene *crb* modulates Aβ42 mediated neurodegeneration. Wild type (A) adult compound eye, a highly organized structure comprising of 750–800 ommatidia [18], which develops from (B, C) eye-imaginal disc. Third-instar eye imaginal disc stained with membrane specific marker, Disc large (Dlg; white), a pan neural marker Elav (red, marks photoreceptors), and (C) Crb protein expression. The Crb expression is localized on the apical surface of epithelial cells and accumulates at the apical membrane's outer margin [51]. (D–F) Misexpression of Aβ42 using GMR-Gal4 driver [22] in the differentiating photoreceptor neurons results in the induction of neurodegeneration as seen in (D) the highly reduced adult eye with a glazed surface and (E, F) eye imaginal disc. Note that in GMR>Aβ42 eye imaginal discs (E) pan neural marker, Elav, exhibits clumping of the photoreceptor neurons and holes in the developing retina, and (F) strong enrichment of Crb expression in the GMR domain. (G–I) Misexpression of Crb full length [41] in GMR>Aβ42 background (GMR>Aβ42+Crb FL) strongly enhances the neurodegeneration phenotype which results in (G) pupal lethality (adults failed to form due to early pupal lethality and as a result lacked the adult eye structure) and (H, I) severe neurodegeneration observed in the eye imaginal disc as evident from (H) fusion of Elav positive photoreceptor neurons, and (I) several fold increase in Crb protein levels. (J–O) Reducing Crb protein levels by using (J–L) *crb^11A22^* allele [33] (GMR>Aβ42+*crb^11A22^*) or (M–O) *crb* RNAi (Vienna Drosophila RNAi Center) (GMR>Aβ42+RNAi) result in the significant rescue of GMR>Aβ42 mediated neurodegeneration as seen in (J, M) the adult eye and (K, L, N, O) the eye imaginal disc. Note that (L, O) the Crb levels are reduced in these backgrounds.

Using the Gal4/UAS system [21], we have developed an AD model with transgenic flies [3] where high levels of Aβ42 are misexpressed in the differentiating photoreceptor neurons of the fly retina using a Glass Multiple Repeat driver [22] (GMR-Gal4>UAS-Aβ42, hereafter GMR>Aβ42). These GMR>Aβ42 transgenic flies exhibit progressive neurodegenerative pathology in the developing retina, which is similar to that observed in AD [3]. Moreover, the misexpression of Aβ42 in the differentiating retina (GMR>Aβ42) exhibits a stronger neurodegenerative phenotype at 29°C [3]. The expression of the cell fate marker like *disc large* (*dlg*, a membrane specific marker) in the developing eye imaginal disc was studied. In comparison to the wild type adult eye (Figure 1A) and the larval eye imaginal disc (Figure 1B), misexpression of Aβ42 (GMR>Aβ42) in the *Drosophila* eye imaginal disc resulted in a reduced eye size with disorganized photoreceptors on the posterior margin as evident from the expression of pan neural marker, Elav (DSHB), in the photoreceptor neurons (Figure 1G), and a highly reduced adult eye which did not show any wild type ommatidium within the compound eye (Figure 1F) [3].

Our earlier studies showed that in the GMR>Aβ42 retina, the ommatidia delaminated from the retinal layers possibly due to loss of polarity and/or cell adhesion [3]. We tested various components of the apical-basal polarity gene pathway in a forward gain of function genetic screen by individually co-expressing the apical basal polarity genes with Aβ42 (GMR>Aβ42+apical basal polarity genes) in the differentiating photoreceptor neurons. From this screen, we identified a transmembrane protein Crumbs (Crb), as a strong genetic modifier of the Aβ42 mediated neurodegenerative phenotype. Crb is highly conserved and has three homologs CRB1, CRB2 and CRB3 in humans. An apical basal polarity gene *crb* encodes Crb protein, which is localized to the apical domain of the epithelial cells, where it is involved in setting up the apico-basal axis of the cell [23]. Furthermore, Crb is required for organizing apico-basal polarity specification, adherens junctions (AJ) and remodeling in epithelial cells [23], [24]. Crb works by forming a complex with Stardust (Sdt/Pals1) [25]. Sdt, in turn, binds to the intracellular domain of Crb and recruits Pals associated tight junction protein (Patj) [26] and Lin7 [27]. To date, Crb has not been reported to play any role in Aβ42 mediated neurodegeneration.

## Materials and Methods

### Fly stocks

All fly stocks used in this study are described in Flybase (http://flybase.bio.indiana.edu). The fly stocks used in this study were GMRGal4>UAS-Aβ42 (GMR>Aβ42) [3], UAS-crb Full Length (II), , UAS- crb^intra^, UAS-crb^intraΔPBM^, UAS- crb^intraΔJM^, UAS-crb^intraΔJMΔPBM^ [28], V39177, V39178 *crumbs* RNAi (Vienna Drosophila RNAi Center) and FRT82B *crb* ^11A22/TM6B^ [23], GMR Gal4 [22].

We have employed Gal4/UAS system for targeted misexpression studies [21]. All Gal4/UAS crosses were maintained at 18°C, 25°C and 29°C, unless specified, to sample different induction levels. The adult fly cultures were maintained at 25°C, while the egg laying (progeny) were transferred to 29°C. Misexpression of Aβ42 in the differentiating retina (GMRGal4>UAS-Aβ42, GMR>Aβ42) exhibits a stronger neurodegenerative phenotype at 29°C [3]. All the targeted misexpression experiments were conducted using the Glass Multiple Repeat driver line (GMR-Gal4), which directs expression of transgenes in the differentiating retinal precursor cells of the developing eye imaginal disc and pupal retina [22].

### Immunohistochemistry

Eye-antennal imaginal discs were dissected from third-instar larvae and stained following standard protocol [29]. Antibodies used were rat anti-Elav (1∶100), rat anti Chaoptin {24B10 (1∶100)}, mouse anti-crumbs (1∶10) (Developmental Studies Hybridoma Bank), rabbit anti-Dlg (1∶200; a gift from K. Cho). Secondary antibodies (Jackson Laboratory) used were goat anti-rat IgG conjugated with Cy5 (1∶200), donkey anti-rabbit IgG conjugated to Cy3 (1∶250), donkey anti-mouse IgG conjugated to Cy3 (1∶200). The tissues were mounted in Vectashield (Vector Laboratories) and immunofluorescent images were captured using the Olympus Fluoview 1000 Confocal Microscope. A modified protocol was used for Crb staining in the eye imaginal disc [30].

### Detection of cell death

Cell death was detected using TUNEL assays from Roche Diagnostics [3], [31]. TUNEL assays were used to identify the cells undergoing cell death where the cleavage of double and single stranded DNA is labeled by a Fluorescein. The fluorescently labeled nucleotides are added to 3′ OH ends in a template-independent manner by Terminal Deoxynucleotidyl transferase (TdT). The fluorescent label tagged fragmented DNA within a dying cell can be detected by fluorescence microscopy. Eye antennal discs after secondary antibody staining [32] were blocked in 10% normal donkey serum in phosphate buffered saline with 0.2% Triton X-100 (PBT) and labeled for TUNEL assays using a cell death detection kit from Roche Diagnostics.

The TUNEL positive cells were counted from five sets of imaginal discs and were used for statistical analysis using Microsoft Excel 2010. The P-values were calculated using one-tailed *t*-test and the error bars represent Standard Deviation from Mean [3].

### Adult eye imaging

Adult eye images were taken on the Axioimager.Z1 Zeiss Apotome. Adult flies were mounted onto a needle and the image was completed by using extended depth of focus function of the Axiovision software version 4.6.3 by compiling the individual stacks from the Z-sectioning approach. The final images and figures were prepared using Adobe Photoshop CS4 software.

## Results

We tested Crb protein levels using Crb antibody (Cq4, DSHB) [23] in the GMR>Aβ42 eye imaginal disc using a modified protocol [30]. The Crb protein is localized to the apical domain of the epithelial cells. We observed higher levels of Crb protein in the GMR>Aβ42 background (Figure 1F) as compared to the wild type eye imaginal disc (Figure 1C). Misexpression of Aβ42 peptide with full length Crb [28] using GMR-Gal4 driver (GMR>Aβ42+Crb (FL), as evident from Crb antibody staining (Figure 1I), resulted in increased neurodegeneration as shown by highly disorganized morphology due to clumping of photoreceptor neurons (Red channel, marked by Elav) of neighboring ommatidia of the eye imaginal disc (Figure 1H). Large gaps were observed among the photoreceptors of the ommatidia where the cells begin to die or clump together. The adults failed to form due to early pupal lethality (Figure 1G). These animals died in the early pupal stages; as a result we could not observe any pupal retina like structures (data not shown). Downregulating Crb levels by using a heterozygous combination of FRT82B *crb^11A22^* allele [33] (Figure 1L) or *crb* RNAi (Figure 1 O) resulted in the rescue of the GMR>Aβ42 mediated neurodegeneration as seen in the eye imaginal disc (Figure 1K, N) as well as in the adult eye (Figure 1J, M). We found significant rescue although complete restoration to the wild type eye was not seen. These results suggested that higher levels of *crbs* are associated with the retina undergoing neurodegeneration due to misexpression of Aβ42. Furthermore, Aβ42 mediated neurodegeneration can be rescued by downregulating *crb* function.

**Figure 2:**
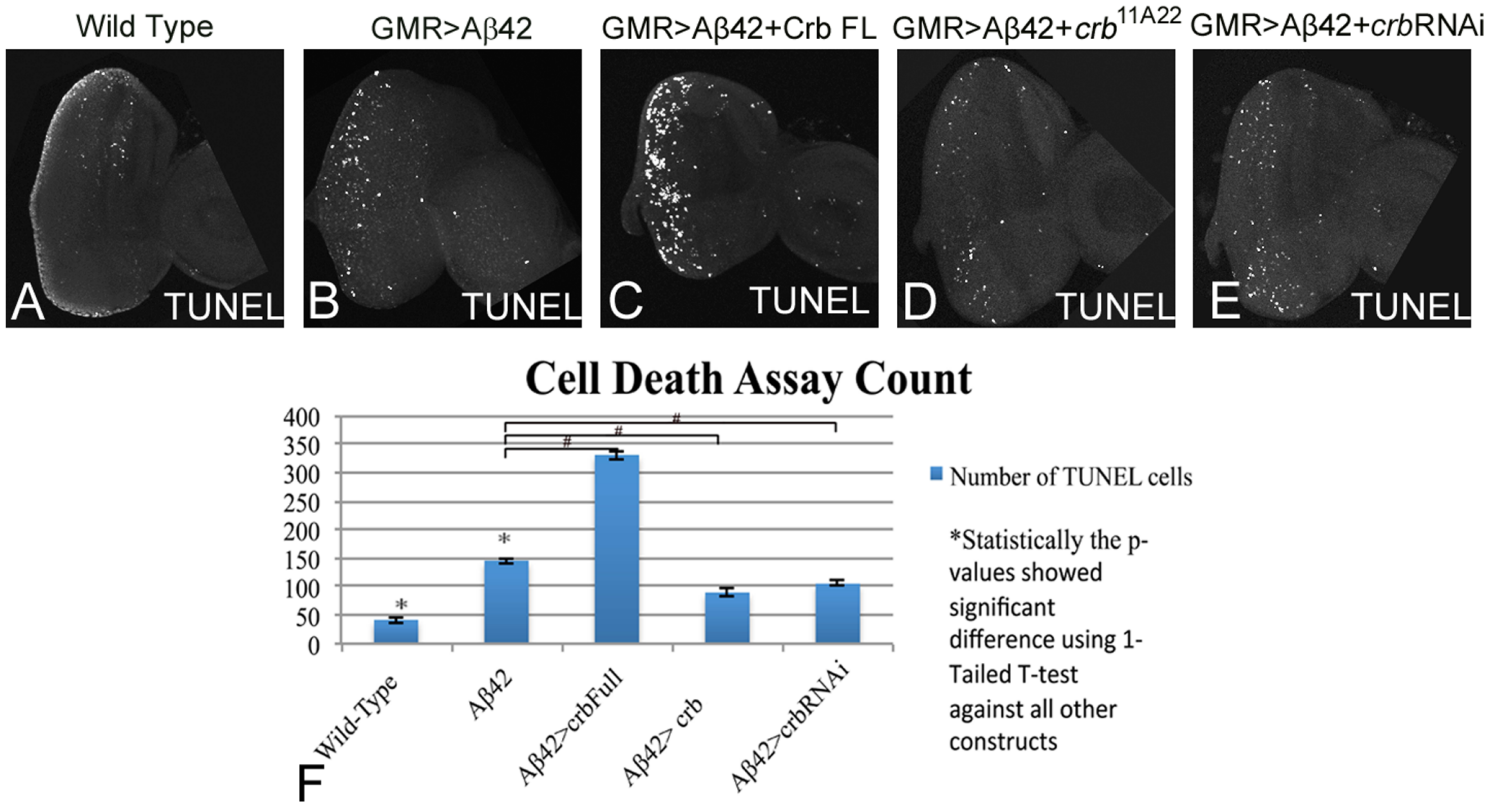
Downregulation of *crb* can block neurodegeneration in the Aβ42 background. TUNEL assays are commonly employed to mark the cells undergoing cell death where the cleavage of double and single stranded DNA is labeled by a Fluorescein [31]. (A) Wild type eye imaginal disc showing a few TUNEL positive nuclei. (B) Misexpression of Aβ42 using GMR-Gal4 driver [22] in the differentiating photoreceptor neurons results in induction of neurodegeneration (B) as seen by a three-fold induction of cell death as evident from number of TUNEL positive nuclei of the dying cells in comparison to (A) wild type eye imaginal disc. Misexpression of Crb full length (FL) in GMR>Aβ42 background (GMR>Aβ42+Crb FL) strongly enhances (C) the neurodegeneration phenotype which results in nearly seven fold increase in number of TUNEL positive nuclei of dying cells in comparison to wild type eye imaginal disc. (D, E) Reducing Crb levels by using (D) *crb^11A22^* mutant allele [33] (GMR>Aβ42+*crb^11A22^*) or (E) *crb* RNAi (VDRC) (GMR>Aβ42+RNAi) result in the rescue of GMR>Aβ42 mediated neurodegeneration as evident from reduction in numbers of TUNEL positive nuclei of the dying cells. (F) Quantitatively, the number of TUNEL cells have been counted and recorded with all five constructs shown. These phenotypes of enhancement of neurodegenerative phenotype and rescue, based on the number of TUNEL positive cells, are significant as seen by the calculation of P-values based on the one-tailed t-test using Microsoft Excel 2010.

We employed TUNEL staining to discern the mechanism of neurodegeneration due to misexpression of Crb in the developing retina. The TUNEL staining marks the nuclei of the dying cells, where the cleavage of double and single stranded DNA is labeled by Fluorescein [31]. Here we utilized TUNEL staining to quantitate the effects of Crb protein levels on neurodegeneration in the GMR>Aβ42 background (Figure 2A–F). The TUNEL positive cells were counted from five sets of imaginal discs and were used for statistical analysis using Microsoft Excel 2010. The P-values were calculated using one-tailed *t*-test and the error bars represent Standard Deviation from the Mean [3]. It is known that a few cells undergo cell death in the wild-type eye imaginal disc (Figure 2A) which does not affect the final morphology of the adult compound eye (Figure 1A). The number of TUNEL positive nuclei of the dying cells in the GMR>Aβ42 flies (Figure 2B) was almost three times as high when compared to the wild-type eye imaginal disc (p = 1.943×10^−6^; Figure 2F). We investigated the levels of Crb with reference to the induction of cell death and found that when Crb levels were increased in a GMR>Aβ42 background (GMR>Aβ42+Crb FL), the TUNEL positive cell number increased (Figure 2C) and was almost seven times higher than the wild type eye imaginal disc (p = 9.536×10^−8^; Figure 2F) and nearly two times higher than the GMR>Aβ42 eye imaginal disc (Figure 2F). Reducing levels of *crb* by using *crb^11A22^* allele [33] (Figure 2D) or *crb* RNAi (Figure 2E) reduced cell death as evident from reduction in the number of TUNEL positive cells to almost two fold with respect to the GMR>Aβ42 eye imaginal disc (for *crb^11A22^* p = 8.386×10^−5^, for *crb* RNAi p = 8.030×10^−5^; Figure 2F).

**Figure 3:**
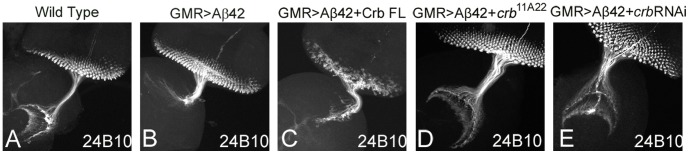
Modulating *crb* levels in the Aβ42 background leads to defects in axonal targeting from retina to the brain. (A) Wild Type eye disc stained with sensory neuron marker, Chaoptin (24B10) [34], which marks only photoreceptor neurons and their axons. The photoreceptor neurons extends through the optic stalk and innervate the medulla and lamina of the larval brain. Note that misexpression of Aβ42 (GMR>Aβ42) in the eye imaginal discs, (B) there is mislocalization of 24B10 expression showing aberrant axonal targeting from retina to brain. The retinal axons fail to innervate the two layers of the brain and end abruptly. (C) Misexpression of Crb full length (FL) in the GMR>Aβ42 background (GMR>Aβ42+Crb FL) strongly enhances the neurodegeneration phenotype which results in (C) lack of axonal targeting from retina to brain. Reducing Crb levels by using (D) *crb^11A22^* allele [33] (GMR>Aβ42+*crb^11A22^*) or (E) *crb* RNAi (VDRC) (GMR>Aβ42+RNAi) result in the significant rescue of GMR>Aβ42 mediated neurodegeneration as evident from the (D, E) restoration of retinal axon targeting.

Next, we investigated the effects of modulating levels of Crb on retinal axon targeting from the retina to the brain using chaoptin (24B10, a marker for photoreceptor cells and their axons [34], DSHB) staining. Disruption of axonal transport mechanisms that leads to axonal vesicle stalling has been shown to contribute to the neurodegenerative phenotypes in the AD fly model [35]. During *Drosophila* visual system development, stereotypical targeting of the axons from the retinal neurons to the special layers of the optic ganglion, medulla and lamina of the brain occurs. The axons of the eight photoreceptor neurons from each ommatidium [36] fasciculate together and project as a single bundle towards the optic lobes of the brain. The *Drosophila* photoreceptors (R cells) seek specific targets to connect in distinct layers of the optic lobes of the brain, *viz.*, R1–R6 axons project to the lamina; R7 and R8 axons project to the separate layers of the medulla [37]. In comparison to the wild-type eye disc where retinal neurons innervate different layers (medulla and lamina) in the brain (Figure 3A), the GMR>Aβ42 eye disc shows complete loss of axonal targeting (Figure 3B). Additional upregulation of full length Crb levels in GMR>Aβ42 (GMR>Aβ42+Crb FL) strongly affected the retinal axon targeting from the retina to the brain (Figure 3C) as compared to the wild type (Figure 3A) and the GMR>Aβ42 alone (Figure 3B). The axonal targeting was restored when *crb* levels were reduced in the GMR>Aβ42 background by using either FRT82B *crb^11A22^* allele (Figure 3D) or *crb* RNAi (Figure 3E). These results further validated our hypothesis that higher levels of Crb enhanced the neurodegenerative phenotype of Aβ42 aggregate accumulation.

**Figure 4:**
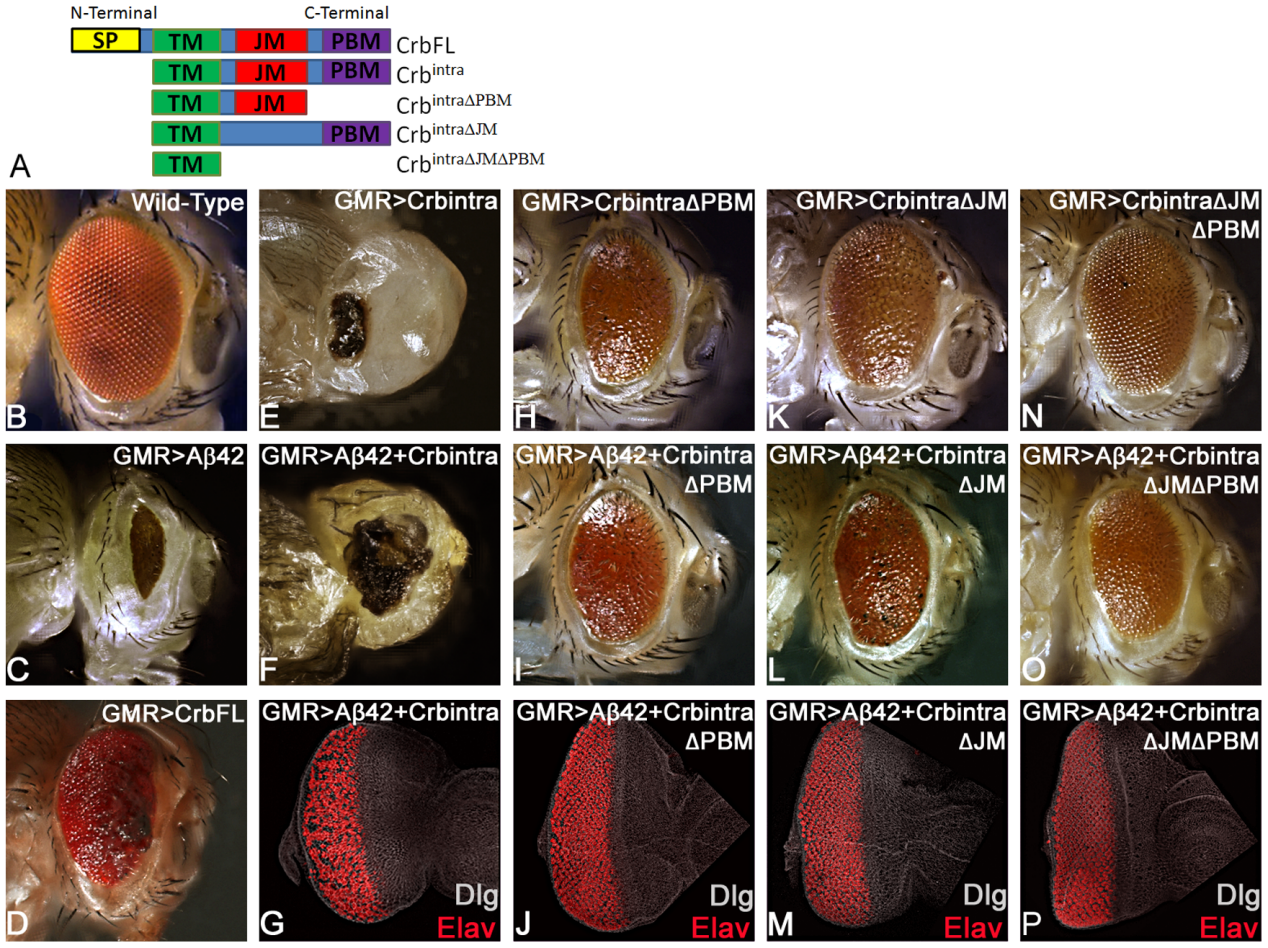
Intracellular domain (ICD) of Crb is required for Aβ42 mediated neurodegeneration. (A) A cartoon depicting full length type I transmembrane Crb protein and various truncated constructs used in this study. The full length Crb protein consists of an extracellular domain (ECD), transmembrane domain (TM), and a short cytoplasmic intracellular domain (ICD), which consists of the juxtamembrane Ferm-binding motif (JM) and PDZ-binding motif (PBM) domains [28]. GMR-Gal4 driver was used for the misexpression studies in the differentiating photoreceptor neurons [22]. (B–D) Adult eyes of (B) Wild-Type, (C) GMR>Aβ42 (GMR enhancer driving overexpression of human Aβ42 in the developing neural retina), and (D) GMR>Crb (FL) are shown as controls. (A, E–F) Misexpression of (E) Crb^intra^ alone, comprising of fully intact ICD, shows a severe phenotype with a small scab on the head cuticle in the adult eye, which is similar to the (F) GMR>Aβ42+Crb^intra^ adult eye. (G) In the GMR>Aβ42+Crb^intra^ eye disc big gaps and holes between photoreceptors of the ommatidia are seen, Dlg (white) marks the membrane and provide an outline of the imaginal disc and pan neural marker Elav [52] marks the photoreceptors. (A, H–P) In the three other Crb constructs, one of the two domains (JM and PBM) of the ICD is either missing or both of them are missing. (H–P) When Crb is missing either (H–J) PBM, or (K–M) JM, or (N–P) both the PBM and JM domain of the ICD, the GMR>Aβ42 neurodegenerative phenotype is restored significantly with the adult eye having a larger size, higher number of ommatidia, and interommatidial bristles. Furthermore, the Elav staining in the eye-imaginal discs shows more organized photoreceptors in comparison to the GMR>Aβ42 eye imaginal disc. (H, K, N) The controls (H) GMR>Crb^intra ΔPBM^, (K) GMR>Crb^intra ΔJM^, and (N) GMR>Crb^intra ΔPBM ΔJM^ showed adult eye phenotypes that are significantly closer to the wild-type. (I, J) When the PBM domain (GMR>Aβ42+Crb^intra ΔPBM^) is missing, (I) the adult eye and (J) the eye imaginal disc showed significant rescue in comparison to the GMR>Aβ42 phenotype. (L, M) When the JM domain (GMR>Aβ42+Crb^intra ΔJM^) is missing, (L) the adult eye and (M) the eye-imaginal disc showed significant rescue in comparison to the GMR>Aβ42 phenotype. (O, P) Finally, when both PBM and JM domains of the ICD are missing (GMR>Aβ42+Crb^intra ΔPBM ΔJM^), a significant rescue was seen in (O) the adult eye and (P) the eye imaginal disc in comparison to the GMR>Aβ42 phenotype.

In order to discern how different domains of Crb protein (Figure 4A) are involved in preventing GMR>Aβ42 mediated neurodegeneration, we used the structure function analysis approach. The full length Crb, a type I transmembrane protein, has 28 EGF domains and four Laminin- AG like repeats in its large extracellular domain (ECD), a transmembrane domain (TM), and a short intracellular domain (ICD) (Figure 4A). The Crb protein's TM domain consists of 37 amino acids spanning the region of the membrane [38]. The ICD contains two motifs, juxtamembrane FERM-binding motif (FBM or JM) domain and C-terminal PDZ (Postsynaptic density/Discs large/ZO-1) binding motif (PBM) domain (Figure 4A). Through its PBM domain, Crb forms a complex with PDZ domain proteins, Stardust and PatJ [25]. It is important to note that the ICD of Crb protein interacts with a variety of conserved proteins including apical basal polarity genes Par6 and aPKC [39], [40]. Prior structure-function studies using the different Crb domains, for example, in the gastrulating embryo, showed that the ubiquitous expression of a membrane-bound cytoplasmic ICD, suppressed the *crb* mutant phenotype to the same extent as full length *crb* [23], [28]. Thus, the different domains of Crb carry out different downstream signaling interactions of the protein, so it is important to investigate which domains are involved in the rescue or enhancement of the neurodegeneration caused by Aβ42.

We employed targeted misexpression [21] of Aβ42 and various domains of Crb protein using the GMR-Gal4 driver [22] for a structure function analysis. The rationale of these studies was to determine which domain of Crb protein is required for its function in Aβ42 mediated neurodegeneration (Figure 1D, E). As discussed previously, in comparison to the wild type eye (Figure 4B), GMR>Aβ42 exhibited strong reduction in size due to neurodegeneration as seen in the adult eye (Figure 4C), whereas GMR>Crb [41] resulted in an increase of the adult eye size with minimal necrosis on the margin (Figure 4D) [42]. Targeted misexpression of Crb ICD (the Crb ICD construct used has been referred to as Crb^intra^ [28]; Figure 4A) in a GMR>Aβ42 background (GMR>Aβ42+Crb^intra^) resulted in strong enhancement of the neurodegenerative phenotype of GMR>Aβ42 alone as seen in the eye imaginal disc (Figure 4G) as well as in the adult eye (Figure 4F). The GMR>Aβ42+Crb^intra^ adult eye showed strong neurodegeneration as evident from the dark necrotic patch in place of the adult eye (Figure 4F). However, the control GMR>Crb^intra^ also showed some neurodegeneration (Figure 4E), which was not as strong as GMR>Aβ42+Crb^intra^ (Figure 4F). Since both the control (Figure 4E) as well as GMR>Aβ42+Crb^intra^ (Figure 4F, G) showed a neurodegenerative phenotype, it raised the possibility of an additive effect. Further experimentation using the truncated constructs of Crb^intra^ domains disproved this additive effect hypothesis. Targeted misexpression of GMR>Aβ42 with Crb^intra ΔPBM^ [28] or Crb^intra ΔJM^ [28] in developing retina resulted in the rescue of the GMR>Aβ42 neurodegenerative phenotype as seen in the eye imaginal disc (Figure 4J, M) as well as the adult eye (Figure 4I, L). The controls GMR>Crb^intra ΔPBM^ (Figure 4H) and GMR>Crb^intra ΔJM^ (Figure 4K) exhibit a slightly reduced adult eye. The Crb^intra^ construct lacking both the JM and PBM domains (GMR>Crb^intra ΔJM ΔPBM^ (Figure 4A)) resulted in a near normal adult eye (Figure 4N). Targeted misexpression of GMR>Aβ42 with Crb^intra ΔJM ΔPBM^ resulted in the rescue of the GMR>Aβ42 neurodegenerative phenotype as seen in the eye imaginal disc (Fig. 4P), and the adult eye (Fig. 4O). All these results clearly demonstrated that like the full length Crb (Crb FL), the entire ICD (Crb^intra^) is also responsible for the enhancement of the neurodegenerative phenotype of GMR>Aβ42. It suggests that Crb ICD is sufficient enough to carry out the Crb FL function in Aβ42 mediated neurodegeneration. When we remove either one or both of the JM and PBM domains from the ICD of Crb, the GMR>Aβ42 phenotype is rescued and the ommatidia are restored to near wild-type. This data strongly indicates that both the JM and PBM domains in Crb are essential to suppress the Aβ42 effects. There might be a correlative interaction between the JM and PBM domains of Crb in the Aβ42 mediated neurodegeneration. However, when we have an intact ICD or full length Crb, there is a severe enhancement of the GMR>Aβ42 phenotype. Also, in the loss-of-function *crb* flies (GMR>Aβ42+*crb^11A22^* and GMR>Aβ42+*crb* RNAi) where we see reduced Crb level expression (Figure 1L, O) as compared to the wild-type (Figure 1C), there is a rescue of Aβ42 mediated neurodegeneration further validating our hypothesis that Crb levels can modify the neurodegenerative phenotype of Aβ42 accumulation. Thus, Crb levels can serve as an excellent biomarker for AD.

**Figure 5:**
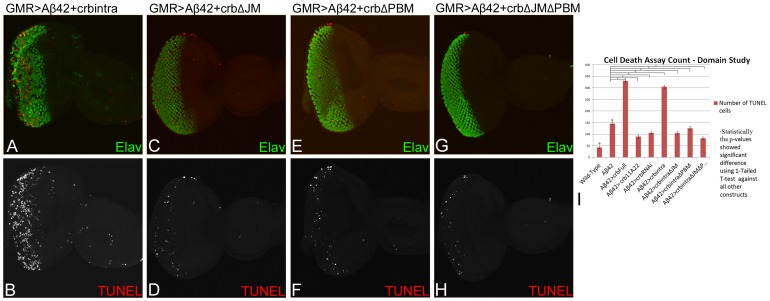
Misexpression of Crb intracellular domain triggers neuronal cell death. (A, C, E, G) The eye-antennal discs are stained with pan neural marker Elav (green), marking the photoreceptor neurons, and TUNEL (red), which marks the nuclei of dying cells. (B, D, F, H) The split channels of the TUNEL cells are shown for better depiction of the TUNEL cells alone. (A, B) In the GMR>Aβ42+crb^intra^ eye disc, the neurodegenerative phenotype of GMR>Aβ42 is enhanced due to increased number of dying photoreceptor neurons as evident from the large number of TUNEL (red) positive cells nuclei, which are (I) calculated quantitatively for all constructs in the bar graph. (A)The dying photoreceptors are clumped and fused together. When we removed either of the PBM, JM or both of these domains within the intracellular domain (ICD) motifs, we see a rescue in the adult eye (Figure 2) and also a (I) decrease in the number of TUNEL positive cells. (C, D) GMR>Aβ42+Crb^intra ΔJM^ (when the JM motif is removed) shows an increase in the (C) organization of the photoreceptors within the ommatidia (Elav) and (C, D, I) a decrease in the number of TUNEL positive cells nuclei as compared to the GMR>Aβ42+Crb^intra^. (D) The number of dying cells in GMR>Aβ42+Crb^intra ΔJM^ is closer to that seen in the wild-type. A similar result was found (E, F, I) when PBM domain was removed from the ICD motif, GMR>Aβ42+Crb^intra ΔPBM^ or (G, H, I) when both the JM and PBM domains were removed from the ICD motif Aβ42+Crb^intra ΔJM ΔPBM^. In comparison to GMR>Aβ42+Crb^intra^, we see a significant decrease in the number of TUNEL positive cells in (E, F, I) GMR>Aβ42+Crb^intra ΔPBM^ and (G, H, I), Aβ42+Crb^intra ΔJM ΔPBM^. (E–H) The number of dying cell nuclei is closer to that seen in the wild-type. Thus, when the ICD is intact (A, B), there is a large number of TUNEL positive cells, which accounts for the adult eye phenotype observed in Figure 2B. However, when either or both of the ICD motifs are removed (C, D, E, F, G, H), there is a significant reduction in the number of TUNEL positive cells as compared to GMR>Aβ42+Crb^intra^ and GMR>Aβ42.

To further verify the structure function analysis results, TUNEL assays were performed on all of the constructs. The rationale was to examine if the reduced eye phenotype seen in GMR>Aβ42+Crb^intra^ was due to cell death or, on the other hand, if the restored eye as shown by removing either or both of the JM and PBM domains of ICD motifs (Figure 4A) is due to reduced number of TUNEL cells. As mentioned earlier, TUNEL marks the nuclei of dying cells, therefore a reduced number of TUNEL positive cells nuclei corresponds to less cells dying, which will lead to a rescue of GMR>Aβ42 neurodegenerative phenotype in the adult eye. We found that the severely reduced adult eye phenotype of GMR>Aβ42+Crb^intra^ is in fact due to an increase in the number of TUNEL positive cells as compared to the wild-type and the GMR>Aβ42 eye disc (Figure 5A, B, I). The GMR>Aβ42+Crb^intra^ exhibits strong neurodegenerative phenotype as evident from disorganized photoreceptor neurons (marked by Elav, green) in the ommatidia. Furthermore, the number of TUNEL positive cells nuclei are increased (Figure 5A, B; red). The TUNEL staining explains the reason for a highly reduced adult eye in GMR>Aβ42+Crb^intra^ (Figure 2F). Additionally, when any either JM or PBM or both JM and PBM domains of the ICD motifs were removed in the GMR>Aβ42 background, the severity of neurodegenerative phenotypes was significantly reduced. In GMR>Aβ42+Crb^intra ΔJM^ (Figure 5C, D), GMR>Aβ42+Crb^intra ΔPBM^ (Figure 5E, F), or GMR>Aβ42+Crb^intra ΔJM ΔPBM^ (Figure 5G, H), the number of TUNEL positive dying cells nuclei were significantly less than GMR>Aβ42 and GMR>Aβ42+Crb^intra^ (Figure 5I). All of these results further validate the data shown in Figure 4 and conforms to the adult eye phenotypes of each of these structures.

For all the ICD motifs of Crb, the TUNEL positive cells were counted from five sets of imaginal discs and were used for statistical analysis using Microsoft Excel 2010. The P-values were calculated using one-tailed *t*-test and the error bars represent Standard Deviation from the Mean [3]. All the p-values showed the TUNEL count to be significantly different from GMR>Aβ42 and the wild-type (Figure 5I). By studying the domains of Crb with reference to the cell death, we found that misexpression of intact Crb ICD domain in GMR>Aβ42 background (GMR>Aβ42+Crb^intra^), resulted in the increased number of TUNEL positive cell (Figure 5I) and was almost six times higher than the wild type eye imaginal disc (p = 1.5559×10^−7^) and nearly two times higher than the GMR>Aβ42 eye imaginal disc (p = 8.7869×10^−^8). Removing the JM motif alone (GMR>Aβ42+Crb^intra ΔJM^ (Figure 5C, D), PBM motif alone (GMR>Aβ42+Crb^intra ΔPBM^ (Figure 5E, F), or by removing both the ICD motifs (GMR>Aβ42+Crb^intra ΔJM ΔPBM^ (Figure 5G, H) resulted in reduced numbers of TUNEL positive dying cells nuclei. The dying cells nuclei in these truncated constructs (Figure 5C–H) were significantly lower than GMR>Aβ42 (for Crb^intra ΔJM^ p = 3.3329×10^−5^, for Crb^intra ΔPBM^ p = 1.5028×10^−5^, for Crb^intra ΔJM ΔPBM^ p = 8.9278×10^−6^; Figure 5I). This TUNEL assay further validated our hypothesis that the reduced eye phenotype seen in GMR>Aβ42+Crb^intra^ (with its fully intact ICD) is primarily due to induction of cell death and the restored eye phenotypes seen when any one or both of the ICD motifs of Crb is/are removed, does indeed have reduced number of dying cells as evident from TUNEL staining.

**Figure 6:**
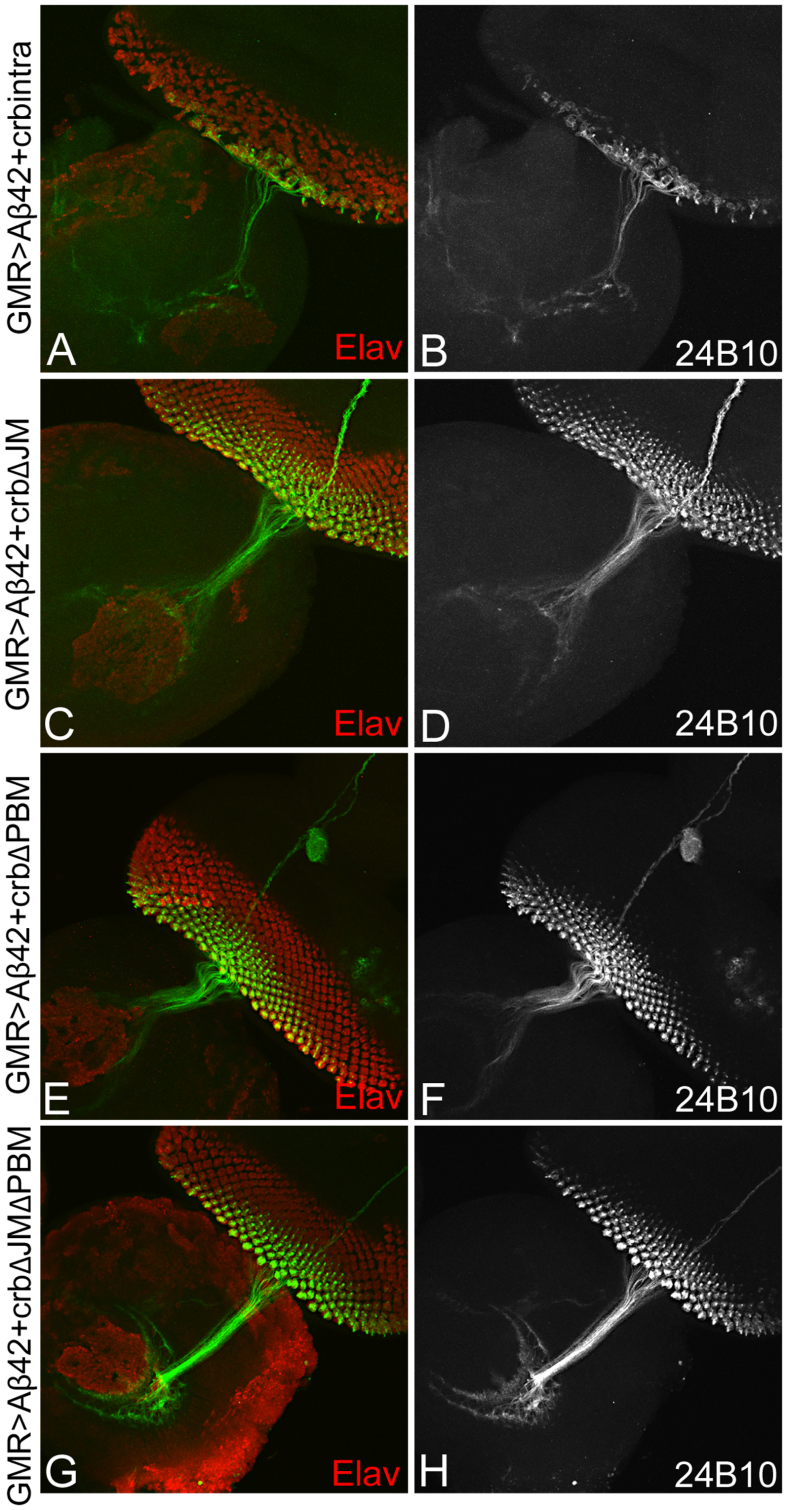
Misexpression of Crb intracellular domain (ICD) can impair axonal targeting. The eye-antennal disc is stained with Elav (red), which marks the photoreceptors, and 24B10 (chaoptin; green), which stains the axons from the retina to the brain [34]. (A, B) Misexpression of intact instar cellular domain ICD GMR>Aβ42+Crb^intra^ results in clumping of photoreceptors (Elav; A), disorganization of axonal targeting from the retina to the brain as evident from 24B10 staining. (C, D)When the JM motif using GMR>Aβ42+Crb^intra ΔJM^, photoreceptor organization as well as the axonal targeting is restored to the wild type. Similarly, removing the (E, F) PBM motif of the ICD using GMR>Aβ42+Crb^intra ΔPBM^, or both PBM and JM domain in GMR>Aβ42+Crb^intra ΔJMΔPBM^, result in restoration of axonal targeting and photoreceptors. Thus, ICD domain of Crb is required for its role in neurodegeneration.

To further test our hypothesis, we looked at the axonal targeting from the retina to the brain using 24B10 (Chaoptin) in these constructs (Figure 4A). As mentioned earlier, 24B10 shows an organized and orderly axon branching from the retina to the brain in the wild-type background (Figure 3A). However, when we observed the 24B10 staining in the GMR>Aβ42+Crb^intra^ eye there is extreme disorganization marked by the clumping of axons, as well as Elav (red) positive photoreceptors which results in impairing of axonal targeting from retina to the brain (Figure 6A, B). This data further confirms our TUNEL data using GMR>Aβ42+Crb^intra^. Additionally, when we analyzed other constructs of Crb by removing either or both of the JM or PBM domains from the ICD motif, there is a rescue of the adult eye (Figure 4A, H–P), a reduction in the number of TUNEL positive (Figure 5C–I), and restoration of the organization of axons from the retina to the brain (Fig. 6 C–H) in all three constructs (GMR>Aβ42+Crb^intra ΔJM^ (Figure 6C, D), GMR>Aβ42+Crb^intra ΔPBM^ (Figure 6E, F), GMR>Aβ42+Crb^intra ΔJM ΔPBM^ (Figure 6G, H). When the JM motif (GMR>Aβ42+Crb^intra ΔJM^ (Figure 6C, D) or the PBM motif (GMR>Aβ42+Crb^intra ΔPBM^ (Figure 6E, F) was removed, there is restoration of the axonal targeting as evident from the 24B10 staining and marking the axonal projections innervate the two layers of the brain. Furthermore, when we remove both of the ICD motifs (GMR>Aβ42+Crb^intra ΔJMΔPBM^ (Figure 6G, H), the axonal connection to the brain is restored to near wild type axonal targeting. These data further validates that the ICD domain of Crb is sufficient enough for Crb function in Aβ42 mediated neurodegeneration.

## Discussion

Our studies strongly suggest that transmembrane protein Crb is involved in Aβ42 mediated neurodegeneration. During wing development, N upregulates *crb* transcription at the dorso-ventral (DV) boundary, and the ability of Crb to inhibit the activity of the γ-secretase complex has been proposed to help refine the N activity domain [43]. Crb functions as a negative regulator of the N signaling pathway [42]. Notch is involved in the development and organization of the dorso-ventral boundary through cell proliferation of the developing eye. Because N and Amyloid Precurssor Protein (APP) are cleaved by similar secretases [44] and Crb regulates N, the Crb effects on Aβ42 could be caused through N regulation. However, in the GMR>Aβ42 model used in our studies, the Aβ42 protein is already cleaved from of APP and does not require cleavage by β- and γ-secretase. Therefore, our data using the transgenic model suggests that Crb also acts downstream of γ-secretase mediated cleavage of APP. Furthermore, higher levels of Crb can enhance human Aβ42 mediated neurodegeneration [3]. Thus, Crb role in modulating Aβ42 mediated neurodegeneration is downstream of N signaling pathway.

In addition, Crb is an upstream regulator of the organ size growth control pathway *viz.*, Hippo signaling pathway. Recently, it was shown that Crb interacts with its juxtamembrane FERM-binding motif (JM) with the FERM domain of Expanded (Ex) to regulate growth by affecting the Hippo pathway activity [45]–[47]. Our structure function analysis studies exhibited that ICD of Crb is sufficient for its role in Aβ42 mediated neurodegeneration suggesting that Crb may act independent of its interaction with Hippo pathway member Ex in Aβ42 mediated neurodegeneration.

Since Crb ICD is involved in its interaction with apical basal polarity gene localization, there is a strong possibility that higher level of Crb in a GMR>Aβ42 background might affect the apical basal polarity of the retinal photoreceptor neurons which result in neurodegeneration. Mutations in Crb homolog 1 (CRB1) has been shown to cause autosomal recessive retinitis pigmentosa (arRP) and autosomal Leber congenital amaurosis (arLCA) [36]. During *Drosophila* eye development, Crb is required in photoreceptors for stalk elongation [42], [48], and in preventing light-dependent retinal degeneration [49]. Mutations in the human Crb homolog (CRB1) result in abnormalities like thick retina and lamination problems [50]. Furthermore, mutant Crb protein is thought to be responsible for retinal degenerations [50]. However, in GMR>Aβ42 background higher levels of Crb protein were responsible for neurodegeneration. Therefore, it is a strong possibility that higher Crb levels may impair apical basal polarity leading to the Aβ42 neurodegeneration. Thus, regulating Crb levels can help prevent the onset of neurodegeneration and Crb may serve as one of the biomarker as well as the key therapeutic targets for the AD.
